# Case of Super-Fœtation

**Published:** 1848-09

**Authors:** A. N. Read


### MEDICAL EXAMINER,

AND

### RECORD OF MEDICAL SCIENCE.

NEW SERIES.—NO. XLV.—SEPTEMBER, 1848.

---

### ORIGINAL COMMUNICATIONS.

---

*Case of Super-Fœtation.* By A. N. Read, M. D.

Extract of a letter from Albert N. Read, M. D., addressed to the editor, dated Andover, Ashtabula county, Ohio, Jan. 4th, 1847.

"Permit me to mention to you one of nature's freaks in generation, which occurred in an adjoining township the past summer. A mare at one birth brought forth a well formed *colt* and a *mule.* She was put to the horse some two or three weeks after having received the Jackass. Both the colt and the mule are doing well. I am aware that the fact of impregnation by different males, is not new to physiologists, but the great difference in time, in this instance, I thought was worth mentioning."

The letter from which the foregoing is extracted, was duly received according to its date, but by one of those accidents to which editors are liable, in common with every body else, it was mislaid. The time which has elapsed, however, although it demands from us this apology to the writer, does not lessen the value of the fact which is related. We shall be glad to hear again from Dr. Read on the subject, and, if it can be ascertained, learn the exact number of days which intervened between the two copulations.—Ed.

